# Experimental absorption corrections for highly absorbing europium compounds in powder neutron diffraction

**DOI:** 10.1107/S1600576726001044

**Published:** 2026-03-08

**Authors:** A. Hoshikawa, K. Kuwahara, C. Sekine

**Affiliations:** a Research and Education Center for Atomic Sciences Ibaraki University Tokai Ibaraki319-1106 Japan; b Graduate School of Science and Engineering Ibaraki University Mito Ibaraki310-8512 Japan; c Graduate School of Engineering Muroran Institute of Technology Muroran Hokkaido050-8585 Japan; Australian Nuclear Science and Technology Organisation, Lucas Heights, Australia

**Keywords:** powder neutron diffraction, high neutron absorption elements, absorption correction

## Abstract

For materials with strong neutron absorption, such as europium (Eu), the absorption correction method is important but difficult to manage. This is because Eu has a strong isotope dependence. The energy dependence of absorption is also complex, making it even more difficult to handle in a time-of-flight neutron diffractometer, where wavelength varies with the time of flight. Experimental measurement of the transmittance of a sample using a vanadium plate, possibly due to unexpected experimental conditions, allowed more accurate structural analysis than absorption correction based on theoretically calculated absorption cross sections.

## Abstract 2

An experimental method for absorption correction of powder samples containing highly neutron absorbing elements is reported.

## 1. Introduction

Neutrons have a different scattering cross section from X-rays, making them an advantageous measurement method for observing light elements around heavy elements and observing magnetic scattering. Furthermore, neutrons have different scattering cross sections and absorption cross sections for each isotope of an element (Sears, 2004 ▸). In particular, some isotopes strongly absorb neutrons and exhibit complex energy dependencies (Murgatroyd & Kelly, 1977 ▸; Kohlmann, 2010 ▸). There have been successful examples of monochromatic neutron diffraction studies of polycrystalline gadolinium compounds using flat-plate single-crystal silicon sample holders (Potter *et al.*, 2007 ▸; Ryan & Cranswick, 2008 ▸). When measuring with a time-of-flight (TOF) neutron diffractometer, it is necessary to consider the energy (wavelength) dependence of absorption on the basis of neutron resonance features (Von Dreele, 2024 ▸). This is critical for absorption corrections in the structure analysis of materials containing such elements with strong absorption.

Previously, we attempted to estimate the absorption cor­rection of EuFe_4_As_12_ (Sekine *et al.*, 2009 ▸) as a polycrystalline europium compound from the integrated intensity of the Bragg reflection using a double cylindrical cell (Oshida *et al.*, 2026 ▸). As a result, it was estimated that the material was ferrimagnetic qualitatively, but a quantitative estimate was difficult. In this work, we measured a powder sample of EuFe_4_As_12_ using a plate-type cell and estimated the wavelength dependence of the absorption factor from incoherent scattering measurements of vanadium. The scattering intensity could be more accurately corrected by using actual measured values rather than the calculated values.

## 2. Wavelength-dependent neutron absorption cross section for Eu

**Figure 1:**
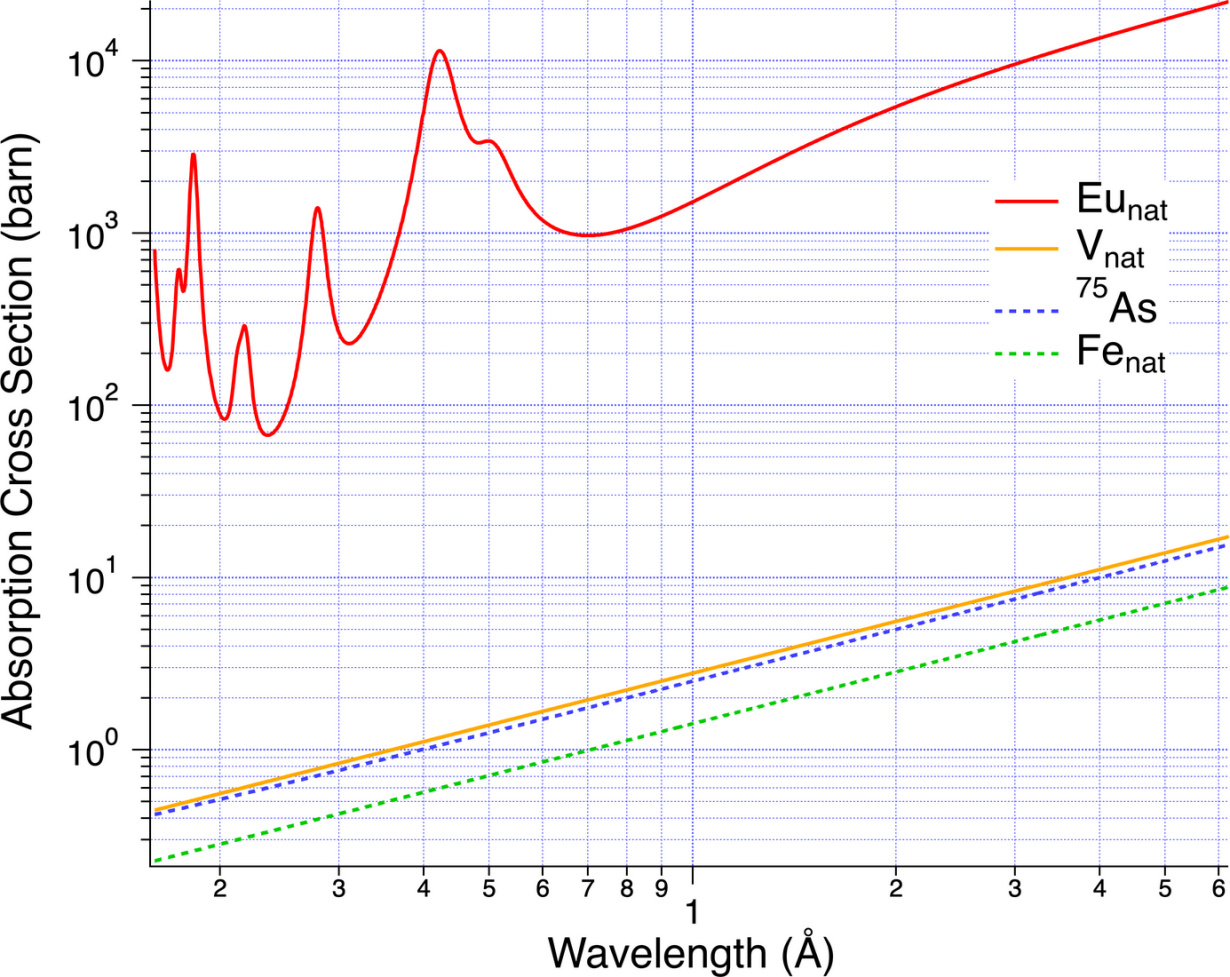
Estimated absorption cross section from the (n, γ) capture cross section of Eu, Fe, As and V using ENDF/B-VIII.1 (Pritychenko, 2025 ▸; Brown *et al.*, 2018 ▸), considering the natural isotope ratio.

Neutrons are scattered or absorbed when they collide with atomic nuclei. In the case of absorption, atomic nuclear reactions such as (n, γ), (n, p) and (n, α) occur. The neutron cross sections are based on a neutron speed of 2200 m s^−1^ (*i.e.E* = 25.3 meV, λ = 1.798 Å) and vary depending on the elements and isotopes. The absorption cross section is usually proportional to 1/*v*, where *v* is the neutron speed. However, some elements, such as Eu, have a strong dependence on energy, and their properties change depending on the isotope (Kohlmann, 2010 ▸). Using the ‘Evaluated Nuclear Data File’ (ENDF/B-VIII.1) library (Pritychenko, 2025 ▸; Brown *et al.*, 2018 ▸; Lynn, 1989 ▸; Lynn & Seeger, 1990 ▸), the (n, γ) capture cross sections for both ^151^Eu and ^153^Eu were estimated as the Eu absorption cross sections by combining them using the isotopic ratios because atomic nucleus reactions dominate the (n, γ) reaction. These capture cross sections are estimated as values relative to the incident neutron energy; we must convert them to the neutron wavelength [*E* (meV) = 81.8/λ^2^ (Å^−2^); Carpenter & Lander, 2004 ▸] at the detector position before use. The isotope ratio of ^151^Eu to ^153^Eu was reported to be 0.478 to 0.522 for natural Eu (Sears, 2004 ▸). Fig. 1 ▸ shows the Eu absorption cross section in the range of neutron wavelength λ from 0.16 to 6.2 Å. Similar estimates were made for iron (^54^Fe: 0.058; ^56^Fe: 0.917; ^57^Fe: 0.022; ^58^Fe: 0.003), arsenic (^75^As: 1.0) and vanadium (^51^V: 0.9975; ^50^V: 0.0025) as these were relevant to the sample used in this study. Only the absorption feature of Eu shows a strong wavelength dependence in Fig. 1 ▸.

## 3. Measurement conditions for the transmittance of the Eu-containing sample

**Figure 2:**
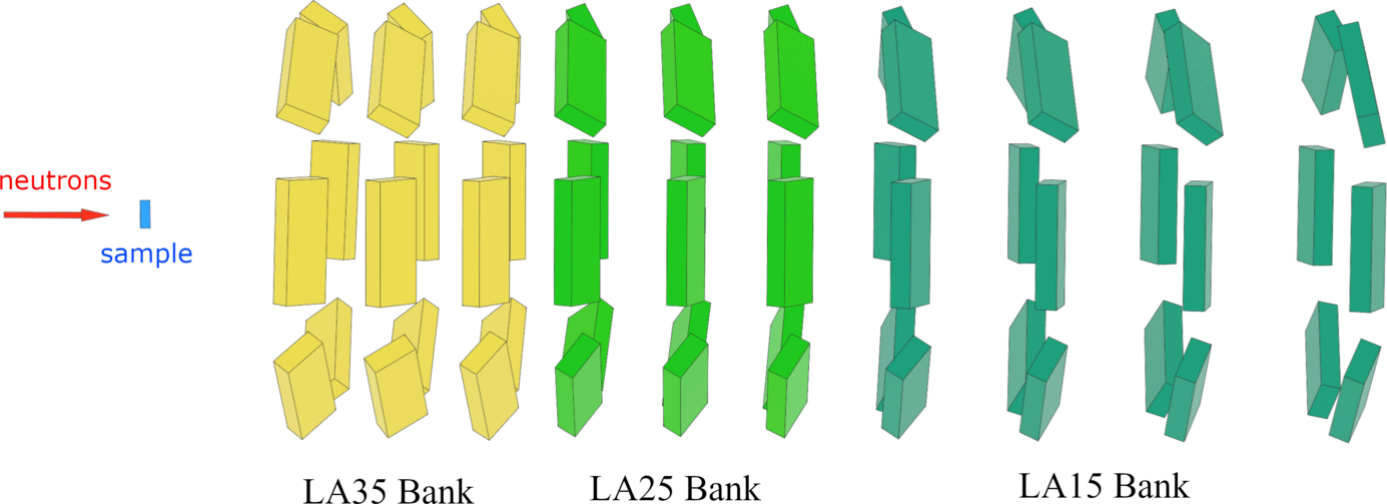
A sketch of the low-angle bank of the TOF diffractometer, iMATERIA. The left side of the figure is the upstream side from which neutrons come after being scattered from the sample. Each rectangular box represents a detector unit. The detectors are mounted on a surface upstream of the detector unit and are positioned so that they do not shadow each other relative to the sample. The figure is divided vertically into ten columns, and the detector units within each column are positioned such that the scattering angle from the sample is equal. The first three columns from the upstream side are grouped as the LA35 bank, the next three columns as the LA25 bank and the remaining four columns as the LA15 bank. The data are summed using time-focusing techniques at each bank.

The measurements were performed at room temperature using the low-angle banks of a TOF-type neutron diffractometer, iMATERIA (Ishigaki *et al.*, 2009 ▸), at J-PARC/MLF in Japan. Each detector is a 12.7 mm-diameter, 64 cm-long tube filled with helium-3 gas. Eight detectors form one detector unit, and the six detector units are arranged in a circular ring so as to have the same scattering angle, as shown in Fig. 2 ▸. Here, 60 detector units have been installed as the low-angle banks. The time-focusing technique is a method for adjusting the TOF to other detectors to match the Bragg peak positions of the detector at a representative scattering angle. The data from the LA15, LA25 and LA35 banks were time-focused according to the TOF to the detector at scattering angles of 15°, 25° and 35°, respectively. The actual wavelength used in this study was estimated at the TOF before time-focusing, considering the central position of the detector unit as a representative time. Here, the TOF differences between pixels within a detector unit and six detector units with the same scattering angle were small and, therefore, ignored as an approximation.

**Figure 3:**
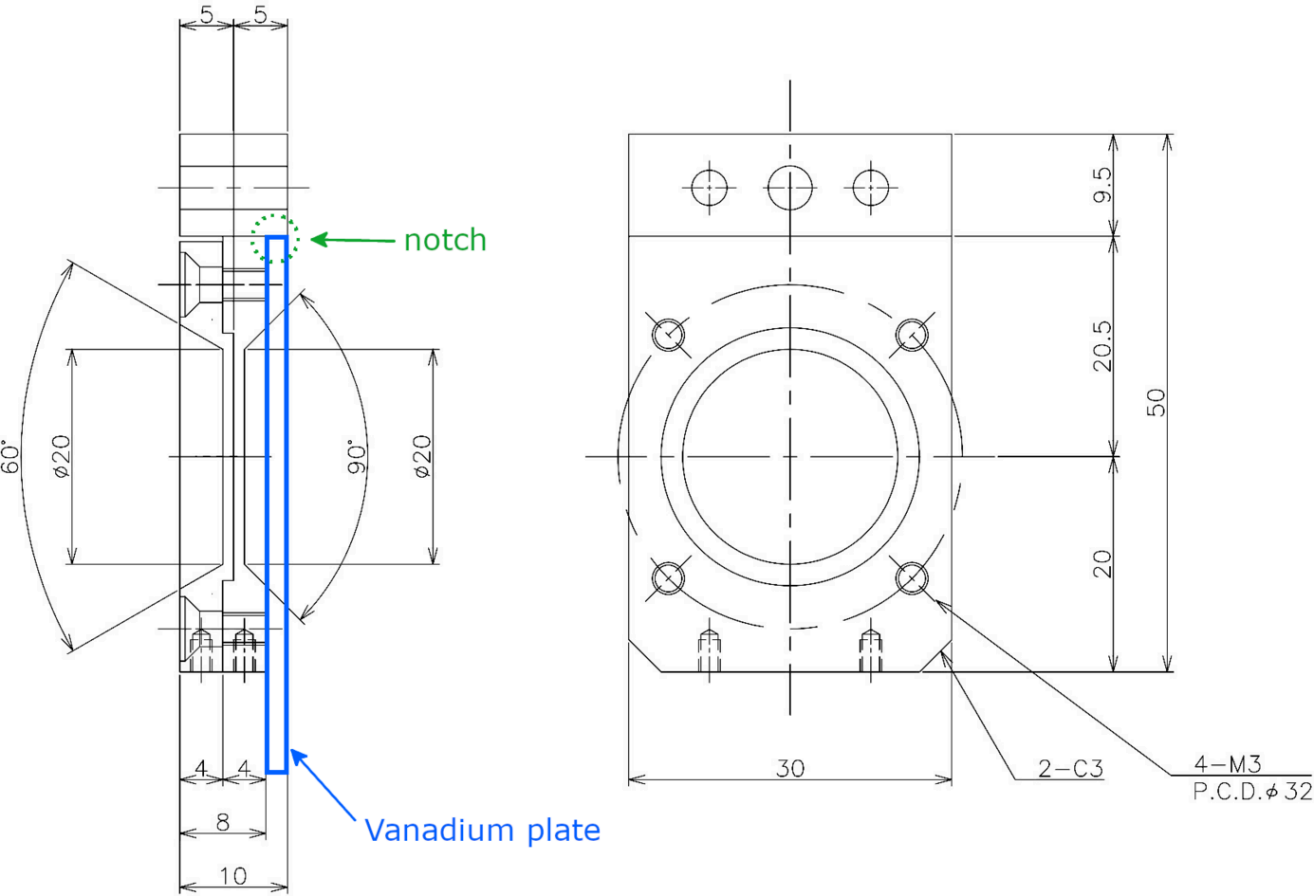
Design of the plate-type cell. The figure on the left represents a cross-sectional view of the central part, while the figure on the right shows a plan view from the downstream side. The holes at the top are designed to secure the cell in an equipment installation position. The 2 mm-thick vanadium plate (thick blue border) was aligned with the notch on the downstream side and secured with tape.

This study used a 0.6 g powder sample of EuFe_4_As_12_ (Sekine *et al.*, 2009 ▸) as a representative Eu sample. It used two plate-type cells with aluminium upstream and downstream windows, 20 mm in diameter and 1 mm thick, as shown in Fig. 3 ▸. The neutron beam size was set to be less than 20 mm in diameter at the sample position, using a 9 mm-diameter aperture at 630 mm upstream from the sample.

One sample cell was filled with the powder sample, and the other was left empty. Absorption measurements were made using incoherent scattering from a 2 mm-thick, 50 mm-square vanadium (V) plate. The V plate was attached to the downstream side of each sample cell and measurements were performed. It was then removed and the main measurements were performed.

## 4. Evaluation of the transmittance

**Figure 4:**
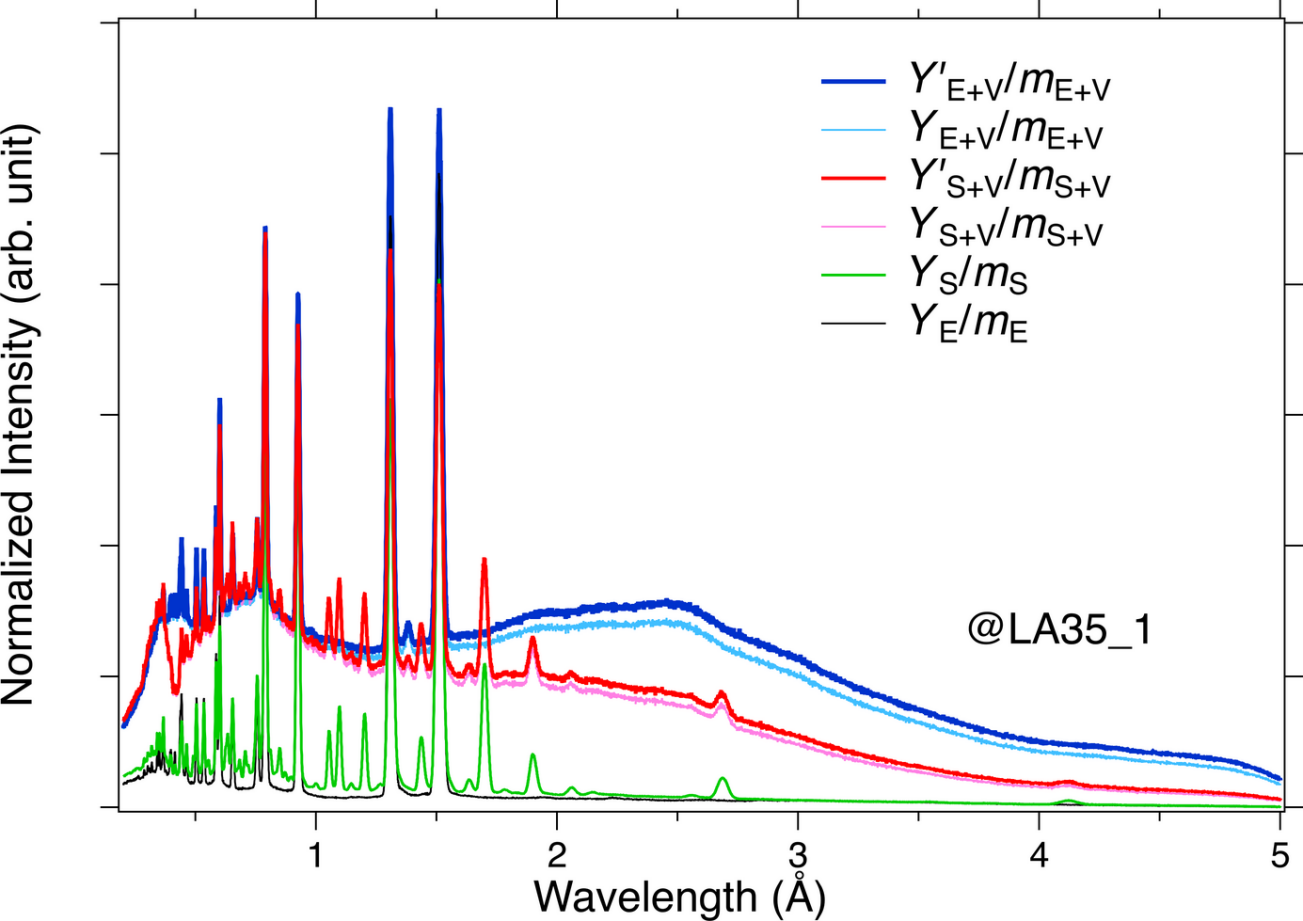
The normalized intensity for the sample and the empty cell, with and without the V plate. These data were observed by the six most upstream detector units (named LA35_1) of the LA35 bank, located on the left side of Fig. 2 ▸, and are shown as representative data.

The transmittance [ϕ(λ)/ϕ_0_(λ), where ϕ_0_(λ) refers to the incident beam and ϕ(λ) the transmitted beam] is estimated from the thickness (*x*) of the plate sample, the absorption cross section [σ(λ)] and the element numbers (*N*) per unit volume of the sample using the following formula: ϕ(λ) = ϕ_0_(λ)exp[−*N*σ(λ)*x*]. Four intensity data sets [*Y_X_*(λ), *X* = S + V, E + V, S, E] were measured: sample + V plate (S + V), empty cell + V plate (E + V), sample (S) and empty cell (E), as shown in Fig. 4 ▸. To compensate for measurement time discrepancies, the intensity was normalized with respect to the monitor counts of the upstream incident beam monitor (*m_X_*, *X* = S + V, E + V, S, E). Here, we converted from TOF (*t*) to wavelength by using the de Broglie relationship, λ = *ht*/*mL*, where *h* is Planck’s constant, *m* is neutron mass and *L* is flight distance. It was necessary to eliminate the absorption effect from the V plate, although the contribution from the aluminium windows was negligible in this regard. We took into account the absorption of the V plate and defined the data 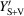
(λ), 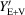
(λ) by dividing by the vanadium transmittance as follows:
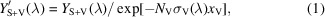

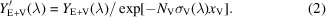
Here, *N*_V_ is the number of vanadium atoms per unit volume, which was estimated from the Avogadro constant, *N*_A_ = 6.02 × 10^23^, the density 6.11 g cm^−3^ and the atomic weight 50.94. σ_V_(λ) is the absorption cross section of vanadium in Fig. 1 ▸, and *x*_V_ is the thickness of the V plate (0.2 cm).

The incoherent scattering from vanadium with and without a sample is defined as *D*_S_(λ) and *D*_E_(λ), respectively, and can be expressed as follows:
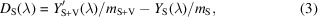

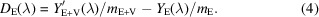


**Figure 5:**
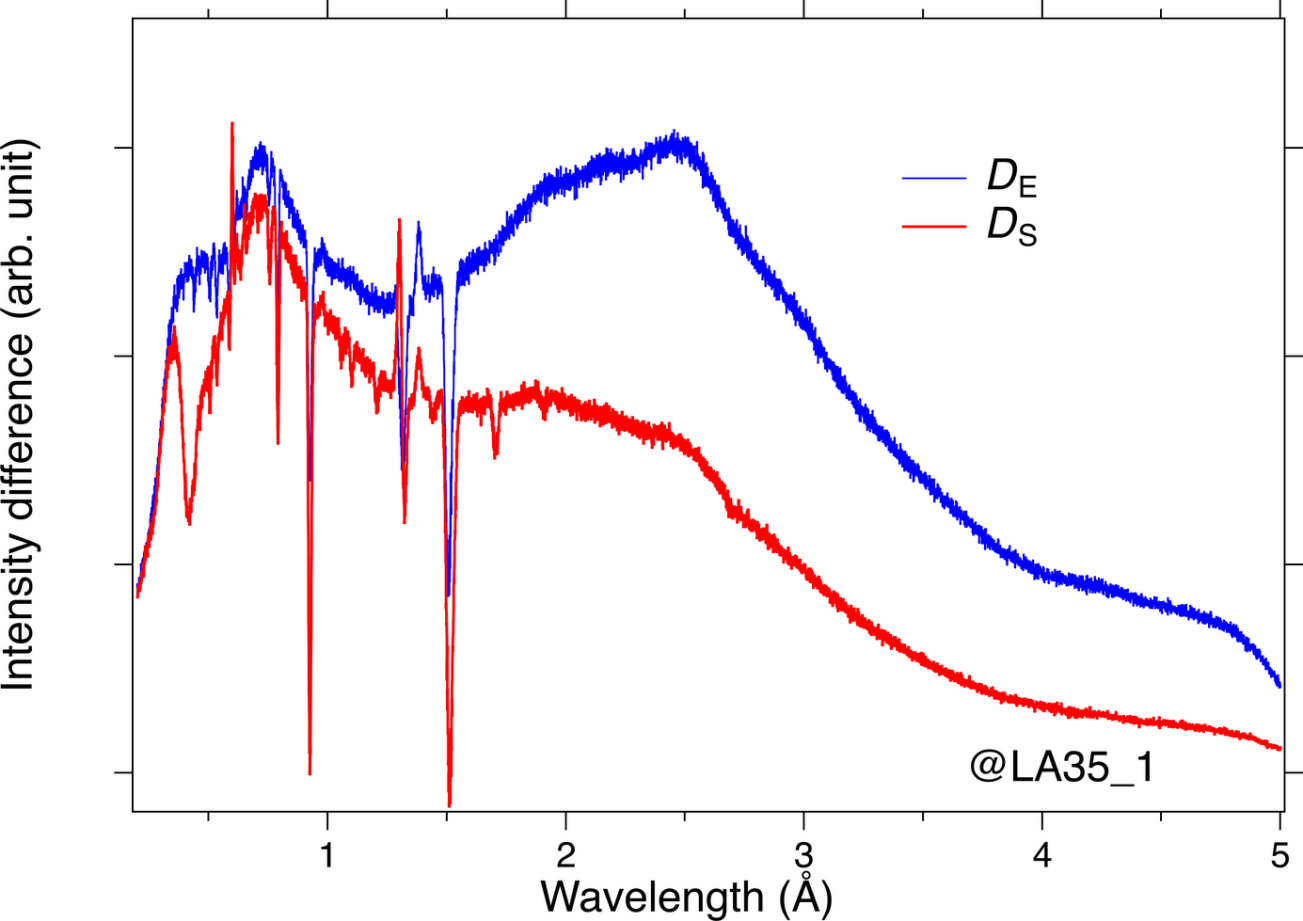
The normalized intensity difference with and without the vanadium plate for the sample and the empty cell. The wavelength dependence of the detector’s detection efficiency and the incident neutron intensity are the same for *D*_S_ and *D*_E_. The sharp peaks and dips correspond to the Bragg peaks from the Al cell and sample.

**Figure 6:**
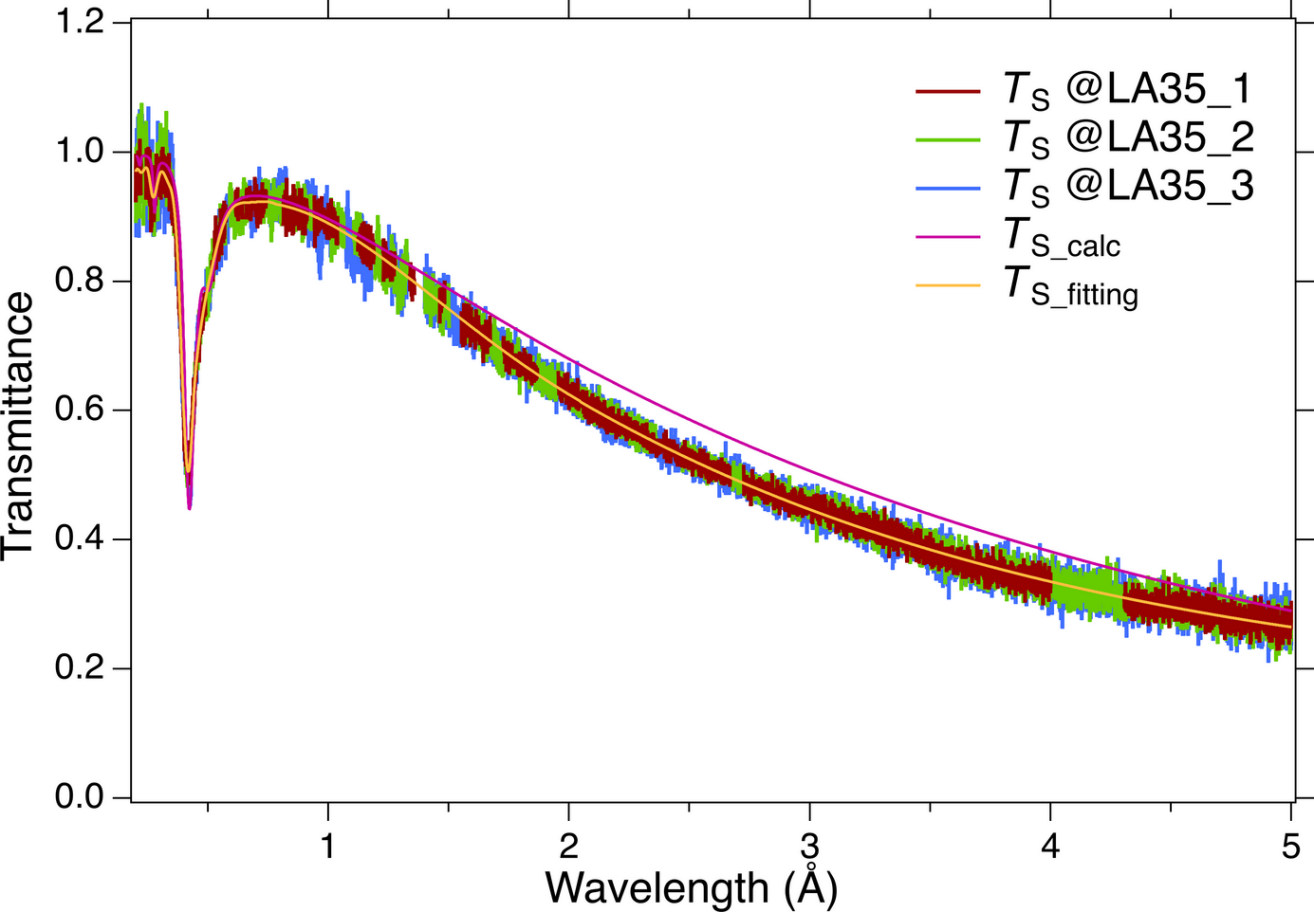
Wavelength dependence of the sample transmittance. Noise due to Bragg peaks has been removed. The *T*_S_calc_(λ) curve is the calculated transmittance from the absorption cross section of Fig. 1 ▸, assuming the sample thickness is 0.045 cm. The *T*_S_fitting_(λ) curve is a curve fitted to reproduce the measured data, *T*_S_(λ), at each detector unit of the LA35 bank. Here, LA35_1, LA35_2 and LA35_3 correspond to the first, second and third columns from the upstream side of the six detector units in the LA35 bank of Fig. 2 ▸, respectively.

As shown in Fig. 5 ▸, *D*_S_(λ) exhibited quite a different behavior from *D*_E_(λ) and strongly reflected the neutron absorption by the sample. The *D*_S_(λ) data show the wavelength dependence of the transmitted beam accompanied by the absorption of the sample. On the other hand, the *D*_E_(λ) data correspond to the wavelength dependence of the incident neutrons. We could estimate the transmittance of the sample [*T*_S_(λ)] as follows:
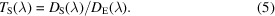


Regardless of the detector location, the sample transmittance [*T*_S_(λ)] shows the same wavelength dependence, as shown in Fig. 6 ▸. Moreover, the wavelength dependence of *T*_S_(λ) was consistent between the LA15 bank and the LA25 bank, despite the different wavelength regions from the LA35 bank. We compared the experimental transmittance with the calculated value from the absorption cross sections. Here, the EuFe_4_As_12_ density and the molecular weight are 3.3 g cm^−3^ and 1274.4, respectively. The total absorption cross section per molecule of EuFe_4_As_12_ is expressed as σ_T_(λ) = σ_Eu_(λ) + 4σ_Fe_(λ) + 12σ_As_(λ). Using these values, the sample thickness required to reproduce the experimental data was estimated to be 0.045 cm. The negative peak in the transmittance around 0.4 Å in Fig. 6 ▸ corresponds to the peak in the absorption cross section of Eu in Fig. 1 ▸ and is found to be in good agreement with the calculated value. As shown in Fig. 6 ▸, the results did not match the experimental results above 1 Å. There appear to be some attenuation effects other than absorption on the long-wavelength side, such as the natural isotope ratio of the sample, detector installation accuracy, time-focusing accuracy, crystal mosaicity and so on. Therefore, the transmittance that reproduces the experimental data was obtained by fitting [*T*_S_fitting_(λ)].

## 5. Crystal structure analysis

Absorption correction was performed using *T*_S_fitting_(λ), and the intensity data were normalized by the incident neutron intensity *I*_0_(λ), which was separately corrected for the vanadium sample, as is typically done with the TOF method, as follows:
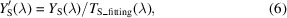

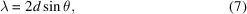

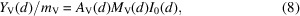

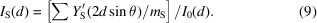
Here 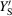
(λ) is the intensity after sample absorption correction, *d* interplanar distance, θ scattering angle, *Y*_V_(*d*) measured vanadium intensity, *m*_V_ monitor counts of the vanadium measurement and *A*_V_(*d*) absorption in the cylindrical vanadium sample (Hewat, 1979 ▸). *M*_V_(*d*) accounts for multiple scattering in the vanadium sample (Blech & Averbach, 1965 ▸). *I*_S_(*d*) is the normalized intensity of the sample as a function of *d* spacing, and this intensity is the data summed across all detector units in the LA35 bank by time-focusing.

**Figure 7:**
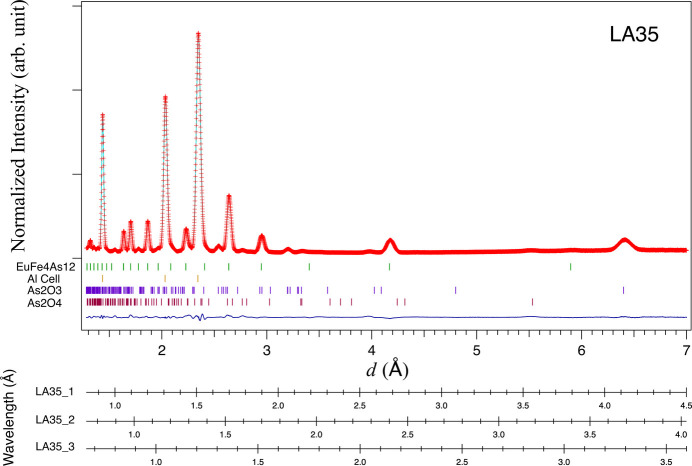
Rietveld analysis of EuFe_4_As_12_ at room temperature. Crosses represent the measurement data that were corrected by *T*_S_fitting_(λ). The calculated data were estimated from the Rietveld method. The tick marks indicate the positions of the Bragg peaks of EuFe_4_As_12_, the Al cell, As_2_O_3_ and As_2_O_4_ from top to bottom. The bottom difference curve is the measured data minus the calculated data. The Rietveld analysis results showed that the mass fractions were EuFe_4_As_12_:As_2_O_3_:As_2_O_4_ = 91.1:8.5:0.4 wt%. The histogram for LA35 is a combination of data from LA35_1, LA35_2 and LA35_3, each with a different wavelength range for the same *d* range. Here, LA35_1, LA35_2 and LA35_3 correspond to the first, second and third columns from the upstream side of the six detector units in the LA35 bank of Fig. 2 ▸, respectively.

The structure analysis for *I*_S_(*d*) was carried out using the Rietveld refinement (Rietveld, 1969 ▸) software *Z-Rietveld* (Oishi *et al.*, 2009 ▸), under conditions without absorption correction, as the absorption correction had already been performed. Some impurity phases, such as As_2_O_3_ and As_2_O_4_, were observed, as shown in Fig. 7 ▸. The Bragg peaks from the Al cell were strong, and we had to consider the preferred orientation. By taking the impurity phases and the Al cell into account, and further excluding the resonance absorption region with wavelengths below approximately 0.7 Å in Fig. 1 ▸, we successfully performed the Rietveld refinement of EuFe_4_As_12_. The structure parameters are shown in Table 1 ▸. In the case of the absorption correction from *T*_S_calc_(λ), the atomic displacement parameters (*B*) became negative, which were not valid results. It was confirmed that correction by *T*_S_fitting_(λ) is necessary for the structural analysis of the sample. This correction is limited to the sample and measurement system used in this experiment. Therefore, it is necessary to make this correction by performing the same measurement with the sample and measurement equipment in each experiment.

## 6. Conclusion

To correct for strong neutron absorption, we typically use the calculated values from the neutron absorption cross sections. However, some elements have complex energy dependencies, and estimating absorption is very difficult. In this study, the sample transmission was experimentally measured from the incoherent scattering from a vanadium plate. We successfully corrected the neutron diffraction patterns for strong absorption and obtained reasonable structural parameters for an Eu compound. The experimentally determined absorption coefficients tend to be larger at longer wavelengths than those estimated by nuclear physics, which is thought to be due to some influences of the experimental environment. Therefore, it is necessary to prepare correction data for each measurement system and make an estimate.

## Figures and Tables

**Table 1:** Atomic coordinates of EuFe_4_As_12_ The space group was *Im*3 (No. 204). The lattice constants were *a* = 8.338454 (2) Å. The reliability parameters (Young, 1993 ▸) of the analysis were χ^2^ = 31.65, *S* = 5.63, *R*_wp_ = 3.804%, *R*_p_ = 2.52%, *R*_e_ = 0.68%.

Atom	*g*	*x*	*y*	*z*	*B* (Å^2^)
Eu	1.0	0	0	0	0.58 (3)
Fe	1.0	1/4	1/4	1/4	0.222 (8)
As	1.0	0	0.34552 (3)	0.15057 (3)	0.129 (7)
